# Understanding Physicians’ Preferences for Telemedicine During the COVID-19 Pandemic: Cross-sectional Study

**DOI:** 10.2196/26565

**Published:** 2021-08-13

**Authors:** Sarah Nies, Shae Patel, Melissa Shafer, Laura Longman, Iman Sharif, Paulo Pina

**Affiliations:** 1 Family Health Centers at New York University Langone Brooklyn, NY United States

**Keywords:** telemedicine, federally qualified health care center, primary care, COVID-19, telehealth, physician, doctor, preference, perspective, dissemination, appropriate, cross-sectional, survey

## Abstract

**Background:**

In contrast to the current broad dissemination of telemedicine across medical specialties, previous research focused on the effectiveness of telemedicine in special populations and for behavioral health encounters, demonstrating that both physician and patient factors impact the efficacious use of telemedicine.

**Objective:**

We aim to evaluate physician perceptions of the appropriateness of telemedicine for patients attending the primary care practices of a federally qualified health center in New York City.

**Methods:**

We used an anonymous cross-sectional survey including closed- and open-ended questions. We used chi-square to test whether providers from certain specialties were more likely to state they would use telemedicine in the future. We used *t* tests to compare age between those who would versus would not use telemedicine. We then used logistic regression to test whether age and specialty were both correlated with the desire to use telemedicine in the future. We used thematic content analysis to describe the reasons providers felt they would not want to use telemedicine in the future and to describe the situations for which they felt telemedicine would be appropriate.

**Results:**

Of 272 health care providers who were sent the electronic survey, 157 (58%) responded within the 2-week survey time frame. The mean age of providers was 45 (range 28-75) years. Overall, 80% (126/157) stated they would use telemedicine in the future. Compared to the family medicine, internal medicine, behavioral health, dental, and obstetrics and gynecology specialties, providers from pediatrics, med-peds, subspecialties, and surgery (protelemedicine specialties) were more likely to believe telemedicine would be useful post pandemic (61/67 [91%] vs 65/90 [72%]; *P*<.001). Providers who reported they would use telemedicine in the future were younger (mean age 44, range 42-46 years vs mean age 50, range 46-55 years; *P*=.048). In the regression analysis, both protelemedicine specialties and age were significantly associated with odds of reporting they would use telemedicine in the future (prospecialties: odds ratio 5.2, 95% CI 1.7-16.2; younger age: odds ratio 1.05, 95% CI 1.01-1.08). Providers who did not want to use telemedicine in the future cited concerns about inadequate patient care, lack of physical patient interaction, technology issues, and lack of necessity. Providers who felt telemedicine would be useful cited the following situations: follow-up visits, medication refills, urgent care, patient convenience, and specific conditions such as behavioral health, dermatology visits, and chronic care management.

**Conclusions:**

The majority of health providers in this resource-limited setting in a federally qualified health center believed that telemedicine would be useful for providing care after the pandemic is over.

## Introduction

Telemedicine instantly became the preferred, and for many *only*, mechanism for health care delivery in New York City during the COVID-19 pandemic [1]. Health care institutions quickly established a variety of strategies to deliver telemedicine services using audio-video or audio only platforms compatible with the Health Insurance Portability and Accountability Act (HIPAA) to provide patient’s access to their providers [2-4].

In contrast to the current broad dissemination of telemedicine across medical specialties, prior research focused on the effectiveness of telemedicine mostly in specific populations [5,6] and for behavioral health encounters [7-10]. Research shows that optimal and efficacious use of telemedicine requires willingness of both the physician and patient to engage on these nontraditional platforms [9]. Physician satisfaction and preference for telemedicine, however, has not been studied as abundantly, especially after the emergence of COVID-19 [10,11]. Although physician personality (ie, judging vs perceiving) and preference for telemedicine demonstrate some correlation, there are few studies on the association between physician age or specialty with physician preference to use telemedicine for clinical practice [11].

This study qualitatively and quantitatively evaluates physician preferences regarding the use of telemedicine for patients in a large federally qualified health system in Brooklyn, New York. We hypothesized that younger physicians and physicians who provide behavioral health services would be more likely to cite telemedicine as an appropriate and preferred modality of care post pandemic. This hypothesis was formed on the assumption that younger physicians would be more familiar with nontraditional technology platforms and the assumption that behavioral health care service does not require physical assessments. With our qualitative data, it is also our hope to explore and to identify any reservations or shortcomings they may have, in efforts to provide insight into the use of telemedicine as an efficient means of providing quality care in the future.

## Methods

### Design

We devised a unique and anonymous cross-sectional electronic survey for this project to collect qualitative and quantitative data. We surveyed health care providers working for a large federally qualified health care system based in Brooklyn, New York that is comprised of 8 primary care practices (medicine, pediatrics, obstetrics and gynecology [OB/GYN], behavioral health), 6 dental clinics, 9 community medicine sites, and 52 school-based health centers. The study was categorized as exempt research by the New York University (NYU) Institutional Review Board.

Beginning in March 2020, providers had the option of using either Webex, Doximity, or MyChart to deliver telemedicine visits to patients. All three of these telemedicine modalities are compliant with HIPAA [3,4]. Webex appointments were scheduled by practice registration staff, and patients were sent emails with instructions on how to log in to the appointment. To use Doximity, providers individually signed up for the service and downloaded the app. They could then send a text message to a patient’s cell phone number asking them to join a video call, or they could directly call the patient’s phone number to conduct an audio-only visit. Anyone enrolled in the patient portal, Mychart, could access the visit through that application. All providers were encouraged to conduct audio-video visits over audio-only visits if possible.

### Survey Procedures

Based on previous studies, we created a brief survey and emailed a survey web link to all providers in May 2020, approximately 2 months into the COVID-19 pandemic in Brooklyn, New York. Providers provided consent and received several reminders to complete the survey over a 2-week period.

In the survey, physicians were asked which telemedicine platforms they used since the beginning of the pandemic in March 2020. They then were asked in the survey to indicate “yes” or “no” to whether they would like to use any of the telemedicine platforms routinely for patient care if the pandemic was over. If the physician responded “yes,” they were asked which of the platforms they would want to use going forward. If the physician responded “no,” they were prompted to explain “why not” and if there were exceptions as to when telemedicine would be useful in patient care. To assess survey consistency, Conbrach alpha was calculated with an acceptable score of .63. To further assess and identify common themes of physician preference, another free writing prompt within the survey asked the physicians to identify what specific patient care situations they felt telemedicine would be most helpful to use.

To assess physician age preference and specialty preference, the survey asked the physician to fill in their age and specialty. The listed specialties included pediatrics, family medicine, internal medicine, med-peds, behavioral health, dental, OB/GYN, and other. If “other” was selected, the participant was asked to describe the specialty.

### Analyses

We tabulated descriptive statistics for all survey participants. Missing data for age was imputed at the mean value. We used chi-square to test whether providers from certain specialties were more likely to state they would use telemedicine in the future. We used *t* tests to compare age between those who would and those who would not use telemedicine. We then used logistic regression to test whether age and specialty were both correlated with desire to use telemedicine in the future. For this analysis, we combined specialties that were more likely to state they would use telemedicine in the future into one binary variable, “pro-telemedicine specialties.”

To better understand the reasons why providers would or would not use telemedicine in the future, we used thematic content analysis to describe the themes from the open-ended responses. All open-ended responses were read and coded separately by two of the authors who then compared notes and, after discussion with the senior author (IS), came to a consensus of thematic groupings.

## Results

Of 272 health care providers who were sent the electronic survey, 157 (58%) responded within the 2-week survey time frame. Demographics and survey responses are shown in Table 1.

There was a statistically significant difference in preference to use telemedicine in the future by specialty. Compared to the family medicine, internal medicine, behavioral health, dental, and OB/GYN specialties, providers from pediatrics, med-peds, medical subspecialties, and surgery (protelemedicine specialties) were more likely to believe telemedicine would be useful post pandemic (61/67 [91%] vs 65/90 [72%]; *P*<.001). Furthermore, med-peds, pediatrics, and medical subspecialties had the highest percentage of “will use telemedicine in the future” with 100% (2/2), 94% (34/36), and 85% (17/20), respectively. OB/GYN and internal medicine had the lowest percentage of “will use telemedicine in the future” with OB/GYN at 50% (4/8) and internal medicine at 63% (17/27).

Providers who reported they would use telemedicine in the future were younger (mean age 44, range 42-46 years vs mean age 50, range 46-55 years; *P*=.048). In the regression analysis, both protelemedicine specialties and younger age were significantly associated with increasing odds of reporting that they would use telemedicine in the future (protelemedicine specialties: odds ratio 5.2, 95% CI 1.7-16.2; younger age: odds ratio 1.05, 95% CI 1.01-1.08).

**Table 1:** Descriptive statistics for the survey sample.

Variable		Participants (N=157)
Age (years), mean (SD)		45.7 (11.5)
**Specialty, n (%)**		
	Pediatrics	36 (22.9)
	Family medicine	26 (16.5)
	Internal medicine	27 (17.1)
	Med-peds	2 (1.3)
	Behavioral health	9 (5.7)
	Dental	20 (12.7)
	OB/GYN^a^	8 (5.0)
	Surgery	9 (5.7)
	Medical subspecialties	20 (12.7)
**Modalities deemed effective (multiple responses allowed; n=309), n (%)**		
	Telephone	70 (22.7)
	Doximity audio + video	82 (26.5)
	Doximity audio only	32 (10.4)
	Webex audio + video	59 (19.1)
	Webex audio only	18 (5.8)
	Mychart audio + video	29 (9.4)
	None Effective	1 (0.3)
	Did not use	18 (5.8)
**Would use in the future (multiple response allowed; n=126), n (%)**		
	Telephone	36 (28.6)
	Doximity audio + video	73 (57.9)
	Doximity audio only	13 (10.4)
	Webex audio + video	41 (32.5)
	Webex audio only	6 (4.8)
	Mychart audio + video	50 (39.7)
	Other (Zoom, Facetime, Whatsapp etc.)	4 (3.2)

^a^OB/GYN: obstetrics and gynecology

There were 22 open-ended responses to the question “why not?” for respondents who said they would not want to use telemedicine once the pandemic is over, citing concerns about inadequate patient care, lack of physical patient interaction, technology issues, and lack of necessity. The responses were divided into thematic grouping, as seen in Table 2. Concerns included the inability “to perform physical examination” and technology issues such as the “time [required] for the provider to connect.” Other statements were focused on the necessary use of telemedicine during a pandemic, as one of the participants said “I will like to have this technology as an option for certain circumstances but not as a routine way to provide patient care.”

**Table 2:** Themes for open-ended responses for why providers would not use telemedicine once the pandemic is over.

Thematic category	Responses (n=22), n
Patient care (diagnosis, vitals, physical exam, labs)	9
Lack of physical patient interaction	6
Technology issues	4
Necessity (use *only* during a pandemic)	2

There were 151 open-ended responses to the question “For which situations do you think telemedicine would be useful?” The responses were categorized under the following themes: follow-up visits, medication refills, urgent care, patient convenience, psychiatric complaints, dermatology complaints, and chronic care management (Table 3). Some situations were specialty specific:

MFM [maternal fetal-medicine] consultations, other consultations that do not require a physical exam. Follow up prenatal visits that do not require a physical exam (i.e. lab review)

Other suggestions were patient population specific, for example:

Patients with mobility issues. Patients who can’t come in easily for different reasons. Patients who frequently No-show. Patients who can’t get transportation easily. Patients who have caregivers with them who can be together.

Other suggestions fit more general clinical management such as:

Follow up [visits] to discuss test results; check in for medications refill requests; all other “I want to speak to my doctor” situations should be routinely “web” appointments and should be billable and compensated as they all take time and effort

Non-annual visits for routine follow up that do not require a physical exam. examples- responses to medication initiation/titration, lab results, medication refills, Diabetes f/u that are less than 3-4 months.

**Table 3:** Thematic coding results for situations believed to be useful for telemedicine after the pandemic is over.

Thematic category	Responses (n=151), n (%)
Follow-up/lab result visits	47 (31)
Medication refill visits	26 (17)
Urgent care/acute symptom triage	24 (16)
Patient convenience (eg, no transportation, includes older adults)	17 (11)
Psych complaints	14 (9)
Dermatology complaints	13 (9)
Chronic care management (patient can self-report HgA_1c_^a^, BP^b^, lifestyle, symptoms)	10 (7)

^a^HgA_1c_: glycated hemoglobin.

^b^BP: blood pressure.

## Discussion

The majority of health providers in this resource-limited setting of a federally qualified health center believed that telemedicine would be useful for providing care after the pandemic is over.

As previous studies have demonstrated [12,13], we also found that older providers are also less likely to prefer telemedicine. Our data show that health care providers older than 60 years were more likely to discontinue use of telemedicine post COVID-19 compared to those younger than 60 years. The reasons for this preference among physicians older than 60 years are unclear and can be an area for further research to identify specific barriers.

Our findings also suggest there is a significant difference among specialty providers in relation to telemedicine preference. The specialty with the highest number of providers willing to continue telemedicine use post COVID-19 was pediatrics. This comes as no surprise considering there is a substantial body of research outlining the benefits and considerations for using telemedicine in the pediatric setting [14,15]. Responses from the qualitative data set indicated *ease of follow-up* as the most common reason for continued use of virtual visits among pediatricians. Conversely, internal medicine had the highest number of providers unwilling to continue its use in the future compared to other specialties. This was surprising because of the focus on history, imaging, and laboratory findings involved in the exam and diagnostic process of internal medicine primary care.

Most past research has focused on patient preference and outcomes of care [9,12,16-21] rather than provider inclination. Studies suggest that telemedicine has been accepted more by patients than by providers [22], with providers citing technological barriers to care provision [13,23-25]. Interestingly, the most commonly cited reason for not continuing use among our cohort, as revealed in the open-ended question responses, was lack of fundamental patient interaction required for health care, such as vitals and certain physical exams. In medicine, providers pride themselves on creating therapeutic relationships based on sitting in the same room with a person. Although there is an intuitive feeling of what a therapeutic relationship feels like, few studies have examined whether and how physical senses (eg, touch or eye gaze) enhance the therapeutic relationship [25]. Without the ability to interact with a patient physically and apply their nuanced senses, physicians in our cohort were less likely to prefer telemedicine as compared to in-person patient interactions. In addition, although the management of several major chronic conditions such as diabetes, heart disease, and chronic obstructive pulmonary disease have been shown to be adequately treated via telemedicine, it would be useful to have follow-up studies that specifically identify which patient populations and diseases physicians found this type of interaction particularly critical for [18,24].

Although the response rate in this survey was strong for a 2-week time frame, the study represents a snapshot in time with data from one system; the preferences of providers in this setting may not be generalizable to other institutions and settings, and they may also evolve over time. Our sample size was small across all specialties and different preferences may be discovered in a larger population. Our data was also limited to physicians within the NYU health care system, and results may reflect the biases of urban communities. Another possibility is that surveyees had differing interpretations of our question regarding willingness to continue telemedicine use in the future. It is possible that opinions would change if it was more precisely worded to be a supplement to a practice rather than an exclusive option as often required during the COVID-19 pandemic. The qualitative data in this study can guide researchers and practice leaders to work with providers to optimize the use of telemedicine in health care going forward, presenting a small silver lining to the COVID-19 pandemic.

Telemedicine as a method to provide patient care has a wide array of implications that can drastically shape the future of health care. Patients have expressed high satisfaction rates when engaging in technology-based health care interactions [9]. Understanding the reservations of medical professionals, based on age and specialty, can lead to improvements that address their concerns and expand the use of telemedicine in practice. The thematic issues described in survey responses of this study can be expanded upon for future research to help deliver more efficient and advantageous telemedicine delivery.

## Abbreviations

HIPAA: Health Insurance Portability and Accountability Act
MFM: maternal fetal-medicine
NYU: New York University
OB/GYN: obstetrics and gynecology
